# Weight loss in patients with severe obesity after bariatric surgery–the potential role of the chrono-nutrition, chronotype and the circadian misalignment: A study protocol of the ChronoWise prospective cohort

**DOI:** 10.1371/journal.pone.0313096

**Published:** 2024-11-08

**Authors:** Joana Rodrigues, Vânia Magalhães, Maria Paula Santos, Cátia Reis, Fernando Pichel, Paulo Soares, Jorge Santos, Sofia Vilela

**Affiliations:** 1 Instituto de Saúde Pública da Universidade do Porto (ISPUP), Porto, Portugal; 2 Faculdade de Medicina da Universidade do Porto (FMUP), Porto, Portugal; 3 EPIUnit, Instituto de Saúde Pública da Universidade do Porto (ISPUP), Porto, Portugal; 4 Laboratório para a Investigação Integrativa e Translacional em Saúde Populacional (ITR), Porto, Portugal; 5 Serviço de Nutrição, Unidade Local de Saúde de Santo António, Porto, Portugal; 6 Centro de Investigação em Atividade Física, Saúde e Lazer (CIAFEL), Faculdade de Desporto da Universidade do Porto, Porto, Portugal; 7 CRC-W, Faculdade de Ciências Humanas, Universidade Católica Portuguesa, Lisboa, Portugal; 8 Instituto de Saúde Ambiental (ISAMB), Faculdade de Medicina da Universidade de Lisboa, Lisboa, Portugal; 9 Instituto de Medicina Molecular João Lobo Antunes (IMM), Faculdade de Medicina da Universidade de Lisboa, Lisboa, Portugal; 10 Serviço de Cirurgia Digestiva e Extradigestiva, Unidade Local de Saúde de Santo António, Porto, Portugal; 11 Unidade Multidisciplinar de Investigação Biomédica (UMIB), Instituto de Ciências Biomédicas Abel Salazar da Universidade do Porto (ICBAS), Porto, Portugal; Federal University of Rio Grande do Sul: Universidade Federal do Rio Grande do Sul, BRAZIL

## Abstract

**Background:**

Despite the potential effectiveness of bariatric surgery in promoting weight loss, a considerable proportion of patients still face the challenge of achieving optimal post-surgery outcomes. The timing of eating, in addition to the content of what is eaten, as well as chronotype and social jetlag (a marker of circadian misalignment), have been implicated in weight regulation. However, the current understanding of these chrono-related behaviours in individuals undergoing bariatric surgery is still scarce. Thus, this study aims to evaluate the role of chrono-nutrition, chronotype, and circadian misalignment in the weight-loss trajectory among individuals living with severe obesity who underwent bariatric surgery.

**Methods:**

The ChronoWise project is a prospective single-centre cohort study designed to follow patients experiencing bariatric surgery at the Santo António Local Health Unit (ULSSA), Porto, Portugal. Participants will be recruited and evaluated at pre-surgery and followed-up over 3 and 6 months after surgery. The baseline evaluation will be conducted face-to-face during the hospital stay and by telephone or video call on the subsequent evaluations, following standard procedures. Data collection includes sociodemographics, food intake, chrono-nutrition behaviours, sleep time behaviour, sleep quality, screen time, physical activity and exercise behaviours, anthropometric measurements, and biochemical parameters. The ‘Munich Chronotype Questionnaire’ will be used to assess chronotype and social jet lag. Chrono-nutrition dimensions will be measured by the ‘Chrononutrition Profile—Questionnaire’ in all evaluations. Weight and height will be self-reported and retrieved from medical registries. trajectories of weight-loss will be obtained.

**Discussion:**

This study will add important evidence on the role of chrono-nutrition, chronotype and social jetlag profile in weight-loss outcomes after bariatric surgery. Identifying novel approaches to change the paradigm of post-surgical weight management towards a tailored treatment aligned with circadian rhythm may be useful to strengthen the existing treatments and improve patients’ response to bariatric surgery.

## Background

The increasing prevalence of obesity at epidemic rates poses a major public health concern globally [1, 2], particularly in the European Region, where obesity, affecting 23% of adults, has been recognized as a major cause of disability and death [3]. Specifically in Portugal, the proportion of adults living with obesity is 21.6% [4], which is alarming. Obesity is associated with an increased risk of morbidity from cardiovascular diseases, metabolic syndrome, diabetes, and cancer [5]. To improve health outcomes, several strategies have been identified, including dieting, physical activity, behavioural interventions, pharmacology, and surgical interventions [6]. Bariatric surgery is the most effective therapy for the treatment of severe obesity, with potential long-term weight loss and decreased obesity-attributable comorbidities [7, 8]. However, weight loss after bariatric surgery varies substantially, with an extensive proportion of patients responding poorly [9].

Bariatric treatment requires lasting lifestyle changes. Thus, current post-surgery approaches prioritize lifestyle modifications, including regular physical activity and dietary adjustments to achieve optimal weight loss and health benefits balancing caloric intake and energy expenditure [10]. However, other dimensions of physical activity and eating habits could be important for successful weight loss after bariatric surgery. The timing and regularity of exercise have recently attracted attention to the impact of a variety of health outcomes. The existing research suggests that maintaining a consistent morning exercise might support weight management among individuals living with obesity [11]. Likewise, mounting evidence also suggests that ‘when’ we eat is as important as ‘what’ we eat to the obesity treatment [12]. This is an emerging research topic known as chrono-nutrition that refers to the relationship between food intake, specifically its timing, and the circadian system clock [13]. Many cyclic endogenous processes follow a circadian pattern such as sleep, feeding, and metabolism. The light-dark cycle is the strongest synchronizer, but the timing and nutritional composition of meals are recognised as also playing a critical role in circadian entrainment and metabolic homeostasis [13–15]. Therefore, the rhythmic coordination of metabolic pathways is physiologically optimized by aligning food intake with the biological day period. However, obesogenic behaviours, such as irregular eating habits, and late eating, predispose individuals to circadian system desynchrony, disrupting homeostasis and thereby leading to metabolic imbalances, which may contribute to an increased risk of adverse cardiometabolic health outcomes, and ultimately cardiovascular diseases [16, 17]. In that way, research in chrono-nutrition emphasizes the importance of synchrony food intake with the endogenous circadian system, which may positively impact weight loss outcomes [18], particularly relevant after bariatric surgery [19]. Furthermore, the altered timing of food intake may be influenced by individual chronotypes [20]. Chronotype is widely referred to a behavioural expression of an individual’s internal circadian clock system, representing their preference or tendency for morningness/earlier sleep timing or eveningness/ later sleep timing [21]. It has been reported that individuals with an evening chronotype commonly experience circadian misalignment since social schedules are commonly designed for early risers. They also tend to accumulate a sleep debt during the week, by waking up with an alarm clock due to social demands, that usually late chronotypes try to compensate by sleeping longer on weekends [21]. This mismatching between biological clock and their social schedule has been termed as ‘social jetlag’, which may result in adverse health outcomes [22–24]. Chronotype and social jetlag were reported to influence weight regulation [20, 24, 25].

Longitudinal studies have already highlighted that certain chrono-related characteristics, such as eating later in the day, having an evening chronotype and experiencing social jetlag, are associated with poor weight loss outcomes after bariatric surgery [26–28]. Nevertheless, the current understanding of these chrono-related behaviours in individuals undergoing bariatric obesity treatment is still limited. Furthermore, the effect of chronotype and social jetlag in the association between chrono-nutrition and weight loss has not yet been established and requires further investigation. The ChronoWise project aims to evaluate the role of chrono-nutrition, chronotype, and circadian misalignment in the weight loss trajectory in a cohort of patients living with severe obesity who underwent bariatric surgery. The specific objectives are: a) To evaluate chrono-nutrition parameters, chronotype and circadian misalignment profile in a sample of pre-bariatric surgery; b) to define trajectories of weight loss at 3 and 6 months of follow-up and associate with chrono-nutrition, chronotype and social jetlag profile.

## Methods

### Study design and setting

The ChronoWise observational study is a prospective single-centre cohort study designed to follow patients undergoing bariatric surgery at the Santo António Local Health Unit (ULSSA), a public hospital of Porto. This study will be carried out by the Institute of Public Health of the University of Porto (ISPUP) and ULSSA, with an expected total duration of 12 months. The duration of the study has been provisionally determined to span from December 2023 through December 2024.

### Ethics

This research will be conducted according to the Ethical Principles for Medical Research Involving Human Subjects established by the Declaration of Helsinki. The study protocol was approved by the ULSSA and School of Medicine and Biomedical Sciences of University of Porto (ICBAS-UP) Ethics Committee (ref. 2023.325_CE) and by the Data Protection Office (DPO). All participants will be asked to provide written informed consent to participate in the study. Participants’ privacy and confidentiality will be ensured during all stages of data collection and processing. Identification data will be kept confidentially in files separated from the remaining data, linked by an identification ID.

### Participants and recruitment

For study participation, a patient in the preoperative period of bariatric surgery must meet the following inclusion criteria: 1) age ≥18 years; 2) first-time bariatric surgery; 3) body mass index (BMI) ≥40.0 kg/m^2^ or BMI ≥35.0 kg/m^2^ with at least one health risk factor, particularly type 2 diabetes, dyslipidemia, obstructive sleep apnea, obesity hypoventilation syndrome, hypertension or osteoarthritis; 4) non-alcoholic, non-drug dependent and without a significant cognitive impairment or psychiatric disorder.

During the hospital stay prior to the surgery, each potential participant will be approached by a research investigator, who will provide information about the study’s objectives and methodology. A participant information sheet has also been developed and will be given to patients, inviting them to take part in the study and explaining the project. In cases of acceptance, participants will be asked to read and sign a duplicated *Informed consent* form. Additionally, consented participants will immediately attend a baseline evaluation. Those who decline to participate, under the condition of oral consent, will be requested to answer a small refusal questionnaire, encompassing questions regarding sociodemographics, chrono-related behaviours, consumption of fruits and vegetables and exercise practice.

Based on the weekly number of first bariatric procedures undertaken at ULSSA, and the potential for follow-up losses, the expected recruitment period for the study is ten months. The recruitment phase and baseline data collection start on 6^th^ of December of 2023 and are anticipated to continue until 30^th^ September of 2024.

### Data collection

Data will be obtained at pre-surgery and on the 3^rd^ and 6^th^ month after surgery by trained interviewers following standard procedures. Baseline evaluation will be carried out by a face-to-face interview during the hospital stay. The follow-up evaluations will occur by telephone or video call interview according to the participant’s preference.

At all-time points, questionnaires will collect data on food intake, chrono-nutrition behaviours, sleep time behaviour, sleep quality, screen time, physical activity and exercise behaviours, anthropometric measurements, and biochemical parameters. The baseline preoperative questionnaire includes additional information on sociodemographics. **Table 1** summarizes the details of the variables assessed at baseline and follow-up evaluations.

**Table 1:** Summary of data collection.

Study variables	Baseline assessment (Face-to-face interview)	Follow-up assessments (Telephone or video call interview)		
		3 months	6 months	
Interview date	✓	✓	✓	
**Sociodemographics**				
Sex	✓			
Birth Date	✓			
Educational level	✓			
Occupation	✓			
Professional status	✓			
Marital status	✓			
Household size	✓			
Household income status	✓			
**Day-to-day Habits**				
Sitting Time	✓	✓	✓	
Screening Time	✓	✓	✓	
Practice of structured exercise (exercise’s type, duration, and timing)	✓	✓	✓	
Chrononutrition Profile Questionnaire	✓	✓	✓	
**Sleep Behaviours**				
Shift work	✓	✓	✓	
Night work	✓	✓	✓	
Munich Chronotype Questionnaire–short form	✓	✓	✓	
**Medication or Supplement Use**				
Frequency and duration of supplements use	✓	✓	✓	
Frequency and duration of medication use	✓	✓	✓	
**Eating Habits**				
Food frequency questionnaire	✓	✓	✓	
**Physical activity level**				
International Physical Activity Questionnaire–short form	✓	✓	✓	
**Sleep**				
Pittsburgh Sleep Quality Index	✓	✓ (online questionnaire)	✓ (online questionnaire)	
**Anthropometric measurements**				
Self-reported weight	✓	✓		✓
Self-reported height	✓	✓		✓
Measured weight	✓			✓
**Clinical data**				
Type of surgery	✓			
Surgery date	✓			
Diagnosed co-morbidities	✓	✓		✓
**Biochemical parameters**				
Glucose	✓			✓
Insulin	✓			✓
Glycated haemoglobin	✓			✓
Triglycerides	✓			✓
High-density lipoprotein cholesterol	✓			✓
Low-density lipoprotein cholesterol	✓			✓
Very low-density lipoprotein cholesterol	✓			✓

#### Food intake and chrono-nutrition

All participants will be required to report their dietary intake, which will be assessed through an adapted version of a validated food frequency questionnaire (FFQ) [29, 30] applied by a trained interviewer. The FFQ comprises a variety of food items and beverage categories, and a question regarding the intake of dietary supplementation will be added. For each item, the average consumption frequency, ranging from never/less than once a month to six or more times per day, will be gathered. Additionally, participants will also indicate whether their typical portion size is equal to, greater than or less than a standard average portion. At baseline and last follow-up assessments, the FFQ will cover the average dietary intake over the previous three months. In a different way, at the first post-operative evaluation, information on food intake will be gathered with reference to the eating habits of the past month. This is because patients must comply with specific dietary guidelines in the first seven weeks after undergoing bariatric treatment, starting subsequently a more non-restrictive diet. Energy and nutrient intake will be calculated by multiplying the frequency of consumption of each food by the selected portion obtaining daily total energy intake (TEI) and macronutrients (i.e., protein, fat, and carbohydrates) as the proportion of TEI, using the Portuguese Food Composition Table [31, 32]. Other indicators of diet quality, such as fruit and vegetables and energy-dense food daily consumption will be included as potential confounders.

The chrono-nutrition dimensions will be measured by the Chrononutrition Profile–Questionnaire [33], consisting of questions fitting behavioural patterns likely to influence the chrono-nutrition profile. This questionnaire covers chrono-related behaviours, including breakfast skipping, largest meal, evening eating and nighttime snacking. In addition, a question on meal frequency was added. For both work/school and free days, data on meal timing will be collected to further determine the eating window (the time between the first and last eating event of the day), evening latency (time between the last meal and sleep onset), eating midpoint (the midpoint between the first and last eating event), and nighttime fasting.

Chrono-nutrition patterns will be analysed separately for work/school days and free days. To capture weekly patterns, a weighted average will be calculated, combining data from weekdays and weekends. Chrono-nutrition behaviours, including breakfast skipping, largest meal, evening eating, evening latency, night eating, and eating window, will be scored based on the cut-offs suggested by Engwall [34]. Each component will be classified as indicative of good, fair, or poor behaviour, resulting in a score of 0, 1, or 2 points, respectively. Furthermore, the sum of each component will yield a global score ranging from 0 to 12 points, with higher values suggesting a poorer chrononutrition status.

#### Chronotype and social jetlag

The short version of the Munich Chronotype Questionnaire (MCTQ) [21, 22], validated for the Portuguese population [35], will be used to assess estimated sleep duration, chronotype and social jetlag over the last 6 weeks.

The MCTQ collects information on an individual’s sleep behaviours, distinguishing between workdays and free days. Considering that sleep on free days is less constrained by social factors, chronotyping using MCTQ is calculated based on the midpoint between sleep on- and offset on free days (midsleep point on free days) with a further correction for calculated sleep debt. The following formulas will be used:

$$Chronotype={MPS}_{freedays}\;if\;{SD}_{freedays}\leq {SD}_{workdays}$$ (1)

$$Chronotype={MPS}_{freedays}-\frac{{SD}_{freedays}-{SD}_{wordays}}{2}\;if\;{SD}_{freedays}>{SD}_{workdays}$$ (2)

Where MPS_freedays_ is the midsleep point on free days and SD_freedays_ and SD_workdays_ represent total sleep duration on free days and workdays, respectively.

Chronotype will be used as a continuous variable and further categorized according to the median or quartile distribution, as recommended by Roenneberg et al [36] and Reis et al [35].

Social jetlag, as a marker of circadian misalignment, will be calculated as the difference between the midsleep point on free days and the midsleep point on workdays [22].

#### Sleep quality

The sleep quality will be assessed using the validated Portuguese version of Pittsburgh Sleep Quality Index (PSQI) [37], a self-rated questionnaire that gathers information regarding the previous month. It consists of 19 questions covering the seven following components: sleep quality, sleep onset latency, sleep duration, sleep efficiency, sleep disturbances, use of sleeping medications, and daytime dysfunction. Each component is scored on a 0 (no difficulty) to a 3 (severe difficulty) scale. The sum of the scores yields a global score, ranging from 0 to 21. A global score of >5 points indicate poor sleep quality [37].

#### Physical activity and exercise behaviours

Physical activity level will be assessed using the Portuguese version of the International Physical Activity Questionnaire (IPAQ)–Short Form [38], which comprises the performance of activities over the past seven days. Information on the frequency (measured in days per week) and duration (time per day) is assessed regarding a set of activities, including walking, sitting, moderate-intensity and vigorous-intensity activities. The data collected will be computerized into metabolic equivalent (MET)-minutes per week, using established formulas, to determine energy expenditure, which is defined as the sum of walking, moderate, and vigorous MET-min per week scores [39]. The total expenditure will be calculated by multiplying individual’s basal metabolic rate (estimated by the Harris-Benedict equations) by the physical activity level.

Additionally, questions concerning the practice of structured exercise will be included. Information on the type of exercise (e.g., Walking, Pilates, Yoga), duration, as well as the period of the day they usually practice it (i.e., morning, lunchtime, early afternoon, late afternoon, at night) will be collected.

#### Sedentary activities and screen time

Behavioural aspects regarding sitting and screen time will be assessed. Participants will be asked to indicate the average time spent sitting (i.e., at a desk, reading, resting, watching television, using a mobile phone, or tablet, or listening to music) on both work/school and free days. In addition to recording the average daily screen time during the work/school and free days, participants will also detail the time of screen usage in the night period, particularly after dinner and before bedtime, and in bed with the lights on or off.

#### Anthropometric and biochemical parameters

Anthropometric measurements, including weight and height, will be retrieved from the medical registries and self-reported. Body mass index (BMI) will be calculated. The post-operative percentage of excess weight loss (%EWL) will be estimated by dividing the weight loss (kg) by the excess weight (the initial weight minus weight at BMI = 25.0 kg/m^2^) and multiplying by 100. Trajectories of weight loss over the follow-up time will be obtained and patients will be further classified as good or poor weigh loss responders according to the %EWL. Thus, patients with EWL≥50% at the last follow-up will be identified as having a good weight-loss response and the remaining as poor weight-loss responders [9].

Fasting venous blood samples will be collected and analysed at the ULSSA, following routine laboratory procedures, including glucose, insulin, glycated haemoglobin, triglycerides, and high-density lipoprotein (HDL-C), low-density lipoprotein (LDL-C), and very low-density lipoprotein (VLDL-C) cholesterol. The Homeostatic Model Assessment insulin resistance (HOMA-IR) will be calculated as ‘glucose (mg/dL) x insulin (μIU/mL)/405’.

#### Other measurements

Participants’ diagnosed comorbidities, including hypertension, type 2 diabetes mellitus, dyslipidaemia, hepatic steatosis, and others, will be recorded. In addition, details regarding the type of bariatric surgery and its date, along with the time of day the surgery was performed, will be gathered.

History of medication and supplementation intake considering the previous three months will be gathered.

### Sample size calculation

The sample size was estimated through the following formula [40]:

$$n=\frac{{\left\{u\sqrt{\left[\pi (1-\pi )\right]}+v\sqrt{\pi_{0}(1-\pi_{0})}\right\}}^{2}}{\left(\pi -\pi_{0}\right)}$$

- π = proportion of interest.
- π_0_ = null hypothesis proportion.
- *u* = one-sided percentage point of the normal distribution corresponding to 100% − the power, which is conventionally 80%.
- *v* = percentage of the normal distribution corresponding to the required (two-sided) significance level of 5% conventionally.

The observed proportion of late eaters among good weight-loss responders is 37% (р = 0.011) according to Ruiz-Lozano et al [26]. Thus, we assumed that approximately 65% of good weight-loss responders will be early eaters. Based on these assumptions:

- π = proportion of early eaters among good weight-loss responders = 0.65.
- π_0_ = 0.5, assuming that there are no differences between the proportion of early and late eaters among good weight-loss responders.
- *u* = 0.84 (value of the standard normal distribution corresponding to the desired level of 80% of power).
- *v* = 1.96 (value of the standard normal distribution corresponding to the significance level [1.96 for a 2-sided test at the 0.05 level]).

The estimated sample size for this study was 85 participants. Taking into consideration a drop-out rate of 20% during the cohort, the sample size was adjusted by multiplying the obtained value by 100/ (100–20) [40]. Thus, the minimum sample size required is 106 participants.

### Statistical analysis plan

Descriptive statistics consisting of mean and standard deviation or median and interquartile range (IQR) will be described for continuous variables. Categorical variables will be reported as frequencies and percentages. The normality of data will be tested using the Kolmogorov–Smirnov test, visual inspection of box-plots or normal probability plots.

The student’s *t*-test for independent samples or the Mann–Whitney test will be performed to compare independent variables, according to the variable’s distribution. Chi-squared test or Fisher’s test will be used to compare the proportion variables. Comparisons throughout the follow-time will be assessed using one-way repeated measures ANOVA test or Friedman test for paired variables, as applicable. When a significant р-value is obtained, Bonferroni post hoc procedures will be used to evaluate pairwise differences.

The %EWL measured longitudinally at baseline and six months post-surgery will be analysed using linear mixed effects over the two post-surgical time points (3 months and 6 months), after controlling for the baseline BMI.

Mixed effects models will be used to evaluate the associations between exposures, such as chrono-nutrition, chronotype and social jetlag profile, and the weight trajectories at 3 and 6 months after undergoing bariatric surgery. To further evaluate the effect of chronotype and social jetlag on the association between chrono-nutrition and weight loss, interaction terms will be included in the models. Potential confounders such as sex, age, education, daily TEI, sleep duration and quality, physical activity, and other variables will be tested.

Data will be analysed with the SPSS IBM Statistical Software and р <0.05 will be considered statistically significant.

## Discussion

Although the potential effectiveness of bariatric surgery in promoting weight loss in patients living with severe obesity, a wide variability in weight-loss outcomes is observed, with a considerable proportion of patients responding poorly [9]. Hence, considering the challenges encountered by many individuals in achieving desirable post-surgical weight loss, there is a need for increased research into the factors associated with successful weight loss, which is of great concern for the improvement of overall health. Chrono-related behaviours, including chrono-nutrition, chronotype and social jetlag may influence post-surgical weight trajectories [26–28]. Moreover, exercising consistently at a set time each day, seems to enhance exercise adherence and aids in weight management for adults living with overweight or obesity [11]. Therefore, alternative approaches focused on the timing of food intake and circadian preferences have attracted attention to tackle the limited success of behavioural strategies targeting lifestyle modifications, which needs additional investigation in people engaging in bariatric treatment. The ChronoWise study will be able to generate useful information to further elucidate the role of chrono-nutrition, chronotype and social jetlag patterns as potential predictors of post-bariatric surgery weight loss outcomes over 3 and 6 months. We also expect to bridge the gap in knowledge regarding the effect of chronotype and social jetlag on the association between chrono-nutrition and weight loss in patients treated with bariatric surgery. Furthermore, it is important to highlight the setting of our study. The hospital in question is a public facility that has been acknowledged as a reference in the surgical treatment of obesity since 2010, hosting the first hospital in the country to perform bariatric surgery [41]. Additionally, before undergoing bariatric surgery, all patients complete a multidisciplinary assessment process, which results from the collaboration of different specialities to examine and educate the patient, with the ultimate goal of improving the success of the intervention. It is also essential for ensuring the physical and psychological well-being of patients prior to the surgery. Nevertheless, the duration of follow-up must be recognized as a limitation of this study. Due to funding constraints, the follow-up will last only six months, which may be insufficient to establish relationships between chrono-nutrition, chronotype and social jetlag patterns and long-term weight loss effectiveness. During this year, we will actively seek further funding to increase the follow-up by at least 12 months. Any modification to this protocol, including the increase of the follow-up period, will be approved by the ethics committee before implementation. Another potential limitation is related to losses to follow-up, which may compromise the research. In that way, to attempt to increase study compliance, follow-up evaluations will be conducted by telephone or online.

Given that our main hypothesis is that eating later during the day, presenting an evening chronotype and having a higher social jetlag impact negatively weight loss trajectories after bariatric surgery, this project can be an important step to move towards evidence-based recommendations. Besides, evaluating the interaction between chronotype and social jetlag exposures with chrono-nutrition dimensions will provide valuable insights into the factors contributing to poor weight loss response and potential weight regain. Therefore, we anticipate that these insights can be used as references in the development of novel therapeutic strategies, that consider caloric intake and macronutrient distribution, along with the optimization of meal timing, to potentiate the current anti-obesity approaches. Raising the awareness of health professionals with regard to chrono-nutrition-related factors may enable the tailored treatment of individuals with obesity, promoting an alignment of their daily activities according to circadian rhythm. Moreover, current physical activity recommendations do not address optimal exercise timing. However, given the accumulating evidence supporting the advantages of maintaining a consistent morning exercise [11], investigating this behaviour within a bariatric sample could strengthen existing guidelines towards an optimization of post-bariatric outcomes. Thus, the incorporation of timing-related concepts in anti-obesity strategies may be beneficial for patients by impact positively weight loss and subsequently associated comorbidities, which contributes ultimately to enhancing patients’ quality of life, as well as for health services by contributing to the increase of the cost-effectiveness of obesity treatment. Thus, our goals align with the United Nations Sustainable Development Goals, aiming to reduce co-morbidities and all-cause mortality associated with obesity [42].

## References

[1] Boutari C, Mantzoros CS. A 2022 update on the epidemiology of obesity and a call to action: as its twin COVID-19 pandemic appears to be receding, the obesity and dysmetabolism pandemic continues to rage on. Metabolism. 2022;133:155217. doi: 10.1016/j.metabol.2022.155217 35584732 PMC9107388

[2] Global, regional, and national age-sex-specific mortality for 282 causes of death in 195 countries and territories, 1980–2017: a systematic analysis for the Global Burden of Disease Study 2017. Lancet. 2018;392(10159):1736–88. doi: 10.1016/S0140-6736(18)32203-7 30496103 PMC6227606

[3] World Health Organization. WHO European regional obesity report 2022: World Health Organization. Regional Office for Europe; 2022.

[4] Oliveira A, Araújo J, Severo M, Correia D, Ramos E, Torres D, et al. Prevalence of general and abdominal obesity in Portugal: comprehensive results from the National Food, nutrition and physical activity survey 2015–2016. BMC Public Health. 2018;18(1):614. doi: 10.1186/s12889-018-5480-z 29747603 PMC5946450

[5] Safaei M, Sundararajan EA, Driss M, Boulila W, Shapi’i A. A systematic literature review on obesity: Understanding the causes & consequences of obesity and reviewing various machine learning approaches used to predict obesity. Comput Biol Med. 2021;136:104754. 34426171 10.1016/j.compbiomed.2021.104754

[6] Baker JS, Supriya R, Dutheil F, Gao Y. Obesity: Treatments, Conceptualizations, and Future Directions for a Growing Problem. Biology (Basel). 2022;11(2). doi: 10.3390/biology11020160 35205027 PMC8869388

[7] Sjöström L. Review of the key results from the Swedish Obese Subjects (SOS) trial—a prospective controlled intervention study of bariatric surgery. J Intern Med. 2013;273(3):219–34. doi: 10.1111/joim.12012 23163728

[8] Nguyen NT, Varela JE. Bariatric surgery for obesity and metabolic disorders: state of the art. Nat Rev Gastroenterol Hepatol. 2017;14(3):160–9. doi: 10.1038/nrgastro.2016.170 27899816

[9] de Hollanda A, Ruiz T, Jiménez A, Flores L, Lacy A, Vidal J. Patterns of weight loss response following gastric bypass and sleeve gastrectomy. Obesity surgery. 2015;25:1177–83. doi: 10.1007/s11695-014-1512-7 25421881

[10] Tabesh MR, Eghtesadi M, Abolhasani M, Maleklou F, Ejtehadi F, Alizadeh Z. Nutrition, physical activity, and prescription of supplements in pre-and post-bariatric surgery patients: an updated comprehensive practical guideline. Obesity Surgery. 2023;33(8):2557–72. doi: 10.1007/s11695-023-06703-2 37389806

[11] Schumacher LM, Thomas JG, Raynor HA, Rhodes RE, Bond DS. Consistent Morning Exercise May Be Beneficial for Individuals With Obesity. Exerc Sport Sci Rev. 2020;48(4):201–8. doi: 10.1249/JES.0000000000000226 32658039 PMC7492403

[12] Garaulet M, Gómez-Abellán P. Timing of food intake and obesity: a novel association. Physiology & behavior. 2014;134:44–50. doi: 10.1016/j.physbeh.2014.01.001 24467926

[13] Tahara Y, Shibata S. Chronobiology and nutrition. Neuroscience. 2013;253:78–88. doi: 10.1016/j.neuroscience.2013.08.049 24007937

[14] Wehrens SMT, Christou S, Isherwood C, Middleton B, Gibbs MA, Archer SN, et al. Meal Timing Regulates the Human Circadian System. Curr Biol. 2017;27(12):1768–75.e3. doi: 10.1016/j.cub.2017.04.059 28578930 PMC5483233

[15] Albrecht U. Timing to perfection: the biology of central and peripheral circadian clocks. Neuron. 2012;74(2):246–60. doi: 10.1016/j.neuron.2012.04.006 22542179

[16] Katsi V, Papakonstantinou IP, Soulaidopoulos S, Katsiki N, Tsioufis K. Chrononutrition in Cardiometabolic Health. J Clin Med. 2022;11(2). doi: 10.3390/jcm11020296 35053991 PMC8780356

[17] Ahluwalia MK. Chrononutrition-When We Eat Is of the Essence in Tackling Obesity. Nutrients. 2022;14(23). doi: 10.3390/nu14235080 36501110 PMC9739590

[18] Garaulet M, Gómez-Abellán P, Alburquerque-Béjar JJ, Lee YC, Ordovás JM, Scheer FA. Timing of food intake predicts weight loss effectiveness. Int J Obes (Lond). 2013;37(4):604–11. doi: 10.1038/ijo.2012.229 23357955 PMC3756673

[19] Cossec M, Atger F, Blanchard C, Jacobi D. Daily Timing of Meals and Weight Loss After Bariatric Surgery: a Systematic Review. Obes Surg. 2021;31(5):2268–77. doi: 10.1007/s11695-021-05278-0 33604863

[20] van der Merwe C, Münch M, Kruger R. Chronotype Differences in Body Composition, Dietary Intake and Eating Behavior Outcomes: A Scoping Systematic Review. Adv Nutr. 2022;13(6):2357–405. doi: 10.1093/advances/nmac093 36041181 PMC9776742

[21] Roenneberg T, Wirz-Justice A, Merrow M. Life between clocks: daily temporal patterns of human chronotypes. J Biol Rhythms. 2003;18(1):80–90. doi: 10.1177/0748730402239679 12568247

[22] Roenneberg T, Pilz LK, Zerbini G, Winnebeck EC. Chronotype and Social Jetlag: A (Self-) Critical Review. Biology (Basel). 2019;8(3). doi: 10.3390/biology8030054 31336976 PMC6784249

[23] Covassin N, Singh P, Somers VK. Keeping Up With the Clock: Circadian Disruption and Obesity Risk. Hypertension. 2016;68(5):1081–90. doi: 10.1161/HYPERTENSIONAHA.116.06588 27620394 PMC5063707

[24] Zhang R, Cai X, Lin C, Yang W, Lv F, Wu J, et al. The association between metabolic parameters and evening chronotype and social jetlag in non-shift workers: A meta-analysis. Frontiers in Endocrinology. 2022;13:1008820. doi: 10.3389/fendo.2022.1008820 36479212 PMC9720311

[25] Ross KM, Graham Thomas J, Wing RR. Successful weight loss maintenance associated with morning chronotype and better sleep quality. J Behav Med. 2016;39(3):465–71. doi: 10.1007/s10865-015-9704-8 26660638 PMC4854772

[26] Ruiz-Lozano T, Vidal J, de Hollanda A, Scheer F, Garaulet M, Izquierdo-Pulido M. Timing of food intake is associated with weight loss evolution in severe obese patients after bariatric surgery. Clin Nutr. 2016;35(6):1308–14. doi: 10.1016/j.clnu.2016.02.007 26948400

[27] Ruiz-Lozano T, Vidal J, de Hollanda A, Canteras M, Garaulet M, Izquierdo-Pulido M. Evening chronotype associates with obesity in severely obese subjects: interaction with CLOCK 3111T/C. Int J Obes (Lond). 2016;40(10):1550–7. doi: 10.1038/ijo.2016.116 27339606

[28] Carvalho AC, Mota MC, Marot LP, Mattar LA, de Sousa JAG, Araújo ACT, et al. Circadian Misalignment Is Negatively Associated with the Anthropometric, Metabolic and Food Intake Outcomes of Bariatric Patients 6 Months After Surgery. Obes Surg. 2021;31(1):159–69. 32728839 10.1007/s11695-020-04873-x

[29] Lopes C, Aro A, Azevedo A, Ramos E, Barros H. Intake and adipose tissue composition of fatty acids and risk of myocardial infarction in a male Portuguese community sample. J Am Diet Assoc. 2007;107(2):276–86. doi: 10.1016/j.jada.2006.11.008 17258965

[30] Willett WC, Sampson L, Stampfer MJ, Rosner B, Bain C, Witschi J, et al. Reproducibility and validity of a semiquantitative food frequency questionnaire. Am J Epidemiol. 1985;122(1):51–65. doi: 10.1093/oxfordjournals.aje.a114086 4014201

[31] Lopes A, Ravasco F, Oliveira L, Dias MG. Tabela da Composição de Alimentos portuguesa: da compilação à atualização. Boletim Epidemiológico Observações. 2023;12(34):22–6.

[32] Instituto Nacional de Saúde Doutor Ricardo Jorge. Tabela de Composição de Alimentos 2023 [Available from: https://portfir-insa.min-saude.pt/.

[33] Veronda AC, Allison KC, Crosby RD, Irish LA. Development, validation and reliability of the Chrononutrition Profile—Questionnaire. Chronobiol Int. 2020;37(3):375–94. doi: 10.1080/07420528.2019.1692349 31760843 PMC7332160

[34] Engwall AC. Development, Validation and Reliability of the Chrononutrition Profile: North Dakota State University; 2019.

[35] Reis C, Madeira SG, Lopes LV, Paiva T, Roenneberg T. Validation of the Portuguese Variant of the Munich Chronotype Questionnaire (MCTQ(PT)). Front Physiol. 2020;11:795. doi: 10.3389/fphys.2020.00795 32760292 PMC7372122

[36] Roenneberg T, Keller LK, Fischer D, Matera JL, Vetter C, Winnebeck EC. Human activity and rest in situ. Methods in enzymology. 2015;552:257–83. doi: 10.1016/bs.mie.2014.11.028 25707281

[37] Gomes AA, Marques DR, Meiavia AM, Cunha F, Clemente V. Psychometric properties and accuracy of the European Portuguese version of the Pittsburgh Sleep Quality Index in clinical and non-clinical samples. Sleep and Biological Rhythms. 2018;16:413–22.

[38] Craig CL, Marshall AL, Sjöström M, Bauman AE, Booth ML, Ainsworth BE, et al. International physical activity questionnaire: 12-country reliability and validity. Medicine & science in sports & exercise. 2003;35(8):1381–95. doi: 10.1249/01.MSS.0000078924.61453.FB 12900694

[39] IPAQ Research Committee. Guidelines for data processing and analysis of the International Physical Activity Questionnaire (IPAQ)-short and long forms. 2005.

[40] Kirkwood B, Sterne J. Calculation of required sample size. London: Blackwells Science Limited. 1988.

[41] Sobrinho IPPC. Estágio na Unidade de Tratamento Cirúrgico de Obesidade num Hospital Universitário. 2023.

[42] United Nations. The Sustainable Development Goals Report 2023: Special Edition. United Nations New York, NY, USA; 2023.

